# Molecular PET and immuno-PET in gastric cancer immunotherapy: current evidence, clinical utilities, and translational barriers

**DOI:** 10.3389/fimmu.2026.1838846

**Published:** 2026-05-01

**Authors:** Qiang Hu, Yuanshui Sun

**Affiliations:** Tongde Hospital of Zhejiang Province Affiliated to Zhejiang Chinese Medical University (College of Integrated Traditional Chinese and Western Medicine Clinical Medicine), Hangzhou, Zhejiang, China

**Keywords:** FAPI, gastric cancer, granzyme B, HER2, immuno-PET, immunotherapy, molecular PET, PD-L1

## Abstract

This mini-review aims to examine how molecular PET and immuno-PET may complement tissue-based biomarkers in gastric cancer immunotherapy by enabling whole-body assessment of target expression, stromal biology, immune effector activity, and treatment-related inflammatory changes. Gastric cancer remains a major global health burden, while immunotherapy is expanding from advanced disease into perioperative and conversion settings. However, clinical benefit remains heterogeneous, and conventional biomarkers such as PD-L1, HER2, and MSI/dMMR do not fully capture spatial and temporal variation across lesions. Molecular PET broadly encompasses tracers targeting metabolism, receptors, stromal components, and immune biology, whereas immuno-PET uses radiolabeled antibodies or related scaffold proteins to visualize target distribution and, in some settings, pharmacodynamic changes *in vivo*. Current evidence in gastric cancer suggests potential roles for HER2-targeted PET, FAPI PET, and granzyme B PET in lesion characterization, stromal phenotyping, early response assessment, and prognostic stratification. However, PD-L1 imaging in gastric cancer remains supported mainly by preclinical data, and much of the checkpoint-imaging literature is extrapolated from non-gastric malignancies. Major barriers include limited gastric-cancer-specific prospective cohorts, heterogeneous tracer formats and imaging time points, lack of standardized quantitative thresholds, and a shortage of outcome-driven studies. Overall, molecular PET and immuno-PET should currently be viewed as promising translational tools rather than routine clinical biomarkers, with their greatest near-term value likely lying in multimodal patient stratification and early pharmacodynamic assessment.

## 1 Introduction

Gastric cancer remains a major global health burden. According to GLOBOCAN 2022, stomach cancer accounted for 968,784 new cases and 660,175 deaths worldwide, ranking among the leading causes of cancer incidence and mortality (1). At the same time, immunotherapy has substantially reshaped the management of gastric cancer and gastroesophageal junction adenocarcinoma. In advanced disease, studies such as CheckMate 649 and KEYNOTE-859 supported the addition of immune checkpoint blockade to chemotherapy in selected populations, whereas MSI-H tumors showed markedly greater sensitivity to PD-1 blockade than unselected cases (2–4). In resectable disease, perioperative immunotherapy has also moved rapidly from exploratory studies into randomized trials, including perioperative pembrolizumab, durvalumab-based regimens, and HER2-directed chemoimmunotherapy strategies (5–10). These studies provide important clinical context for the expanding role of immunotherapy in gastric and gastroesophageal junction adenocarcinoma, but they do not constitute molecular imaging evidence.

Despite this therapeutic progress, benefit remains uneven across patients and across lesions within the same patient. Current clinical decision-making still relies mainly on tissue-based biomarkers such as PD-L1, HER2, and MSI/dMMR. However, gastric cancer is characterized by marked spatial and temporal heterogeneity, and a single biopsy often fails to represent whole-body target expression, lesion-to-lesion immune variation, stromal context, or treatment-induced biological adaptation. This limitation is particularly relevant in metastatic and perioperative settings, where clinicians increasingly need to determine not only whether immunotherapy is active in principle, but also which patients are likely to benefit, which lesions are biologically sensitive, whether early treatment effects are occurring, and whether resistance or toxicity is emerging.

In this context, molecular PET and immuno-PET are increasingly attractive. Unlike conventional imaging, which mainly depicts anatomy or bulk metabolic change, these approaches can noninvasively interrogate tumor and immune biology throughout the whole-body disease burden. Their potential roles include lesion-level target mapping, characterization of the tumor microenvironment, early functional response evaluation, pharmacodynamic monitoring, and, potentially, support for future theranostic selection. This mini-review therefore summarizes the current evidence, clinical utilities, and major translational barriers of molecular PET and immuno-PET in gastric cancer immunotherapy.

## 2 Molecular PET and immuno-PET: core utilities, distinctions, and tracer design considerations

Molecular PET is a broad concept that includes PET tracers interrogating tumor or immune biology beyond anatomy, such as probes of metabolism, receptor expression, stromal activation, immune-cell function, and treatment-related inflammation (11, 12). Depending on the target and clinical question, molecular PET may serve diagnostic, prognostic, predictive, or response-monitoring functions.

Immuno-PET is a major subcategory of molecular PET. It uses radiolabeled antibodies, antibody fragments, nanobodies, or related scaffold proteins to visualize target distribution *in vivo* (11). Compared with small-molecule tracers, immuno-PET offers high target specificity and is particularly suited to questions involving therapeutic antibodies, immune checkpoints, or dynamic target biology. In selected contexts, immuno-PET can also provide information on drug biodistribution, target engagement, disease phenotyping, and pharmacodynamic change during treatment (11, 12). In practice, intact antibodies are used more commonly in preclinical xenograft studies because of their strong target specificity and longer circulation time, whereas clinical imaging increasingly employs a broader range of tracer formats, including antibody fragments, nanobodies, and smaller scaffold-based probes, to improve imaging kinetics and workflow feasibility.

**Figure 1:**
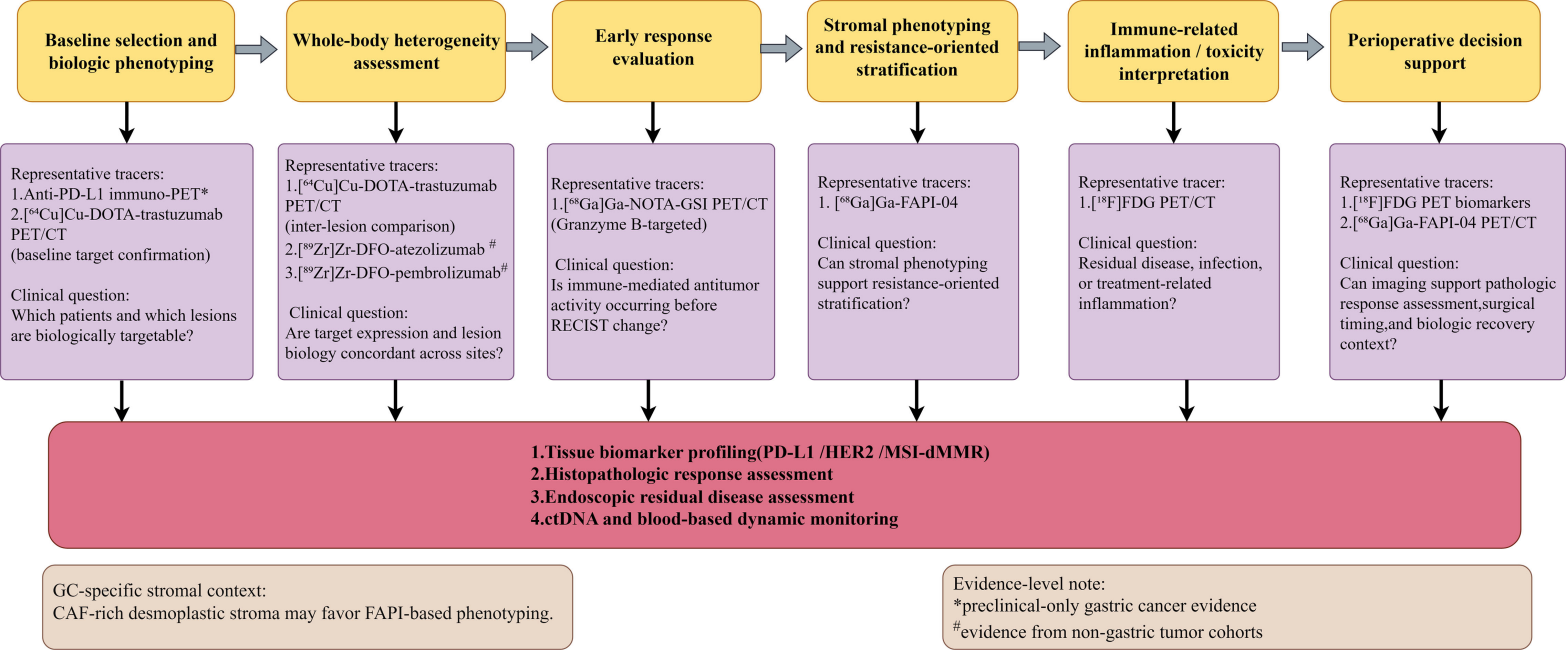
Proposed roles of molecular PET and immuno-PET across the gastric cancer immunotherapy pathway. CAF, cancer-associated fibroblast; ctDNA, circulating tumor DNA; FDG, fluorodeoxyglucose; FAPI, fibroblast activation protein inhibitor; GC, gastric cancer; RECIST, response evaluation criteria in solid tumors.

Tracer design is central to clinical feasibility. Intact antibodies are commonly paired with longer-lived radionuclides such as zirconium-89 because of their slower pharmacokinetics, whereas smaller proteins or low-molecular-weight tracers are more compatible with shorter-lived isotopes such as gallium-68, fluorine-18, or copper-64. These choices directly influence image timing, tumor-to-background contrast, radiation exposure, and workflow practicality. In gastric cancer, where lesions may be peritoneal, multifocal, or adjacent to physiologic abdominal uptake, such format-dependent trade-offs are particularly important. Thus, the question is not simply whether a tracer can bind its target, but whether it can do so with sufficient contrast and within a clinically useful time window (**Figure 1**).

## 3 Why molecular PET and immuno-PET are urgently needed in gastric cancer immunotherapy

The clinical need for molecular PET and immuno-PET in gastric cancer immunotherapy arises from four recurring problems. First, baseline biomarkers remain incomplete: PD-L1 expression is heterogeneous, HER2 can be discordant across lesions and may change during treatment, and MSI-H identifies only a minority of patients. Second, conventional anatomical response assessment may lag behind biological change, particularly after immunotherapy-based combinations. Third, stromal and immune-excluded phenotypes may drive resistance even when tumor-cell targets remain detectable. Fourth, clinicians increasingly need better tools to judge mixed response, focal progression, and perioperative readiness after neoadjuvant immunotherapy.

Some of these limitations are hinted at by conventional metabolic imaging. In a retrospective analysis of 64 gastric cancer patients, Chen et al. found that higher FDG uptake was associated with PD-L1 expression and PD-L1-positive tumor-infiltrating lymphocytes, suggesting that metabolic imaging can partly reflect immune-related tumor biology (13). However, FDG is neither target-specific nor sufficient for individualized immunotherapy decision-making.

More direct evidence for target heterogeneity comes from PD-L1 imaging and should be presented cautiously. Ibrahim et al. demonstrated dynamic and spatially heterogeneous PD-L1 expression in gastric cancer using NCIN87 cell lines, xenograft models, and patient-derived xenograft tissue analyzes (14). This study provides biological support for PD-L1-directed immuno-PET in gastric cancer, but it is preclinical rather than a human gastric cancer PET trial. Therefore, checkpoint imaging in gastric cancer should currently be framed as an emerging translational concept rather than an established clinical tool.

A similar need exists for HER2-directed imaging. Hernandez et al. conducted a pilot study of [^64^Cu]Cu-DOTA-trastuzumab PET/CT in gastric adenocarcinoma and showed that lesion-level HER2 imaging was feasible, although dose optimization, image timing, and lesion detectability remained imperfect (15). Together, these studies suggest that molecular PET and immuno-PET are needed because tissue sampling alone cannot adequately capture the biologic complexity of gastric cancer under immunotherapy.

## 4 What molecular PET and immuno-PET can reveal in gastric cancer

### 4.1 Whole-body target expression and lesion-level heterogeneity

A major promise of molecular PET and immuno-PET is the move from single-site pathology to whole-body target mapping. In gastric cancer, this is especially relevant for PD-L1 and HER2. Whereas biopsy reflects one lesion at one time point, imaging can reveal target-positive lesions across the disease burden, show whether uptake is homogeneous, and identify divergence between primary and metastatic sites.

For PD-L1, gastric-cancer-specific evidence remains limited and largely preclinical. Ibrahim et al. showed that PD-L1 expression in gastric cancer models was dynamic, spatially heterogeneous, and influenced by glycosylation, which altered antibody recognition and imaging performance (14). This work should therefore be regarded as mechanistic support for PD-L1 immuno-PET rather than clinical proof of patient-level utility. Brown et al. likewise emphasized that antibody format, tracer kinetics, and target accessibility are key determinants of PD-L1 imaging performance and are therefore highly relevant to gastric cancer translation (16).

For HER2, early clinical translation has begun. Hernandez et al. showed that [^64^Cu]Cu-DOTA-trastuzumab PET/CT was feasible in gastric cancer patients, although performance remained limited by technical and biologic factors (15). In principle, such whole-body HER2 imaging may help address interlesional heterogeneity, target loss during therapy, and discordance between archival tissue and current lesion status.

Because gastric-cancer-specific checkpoint imaging remains limited, evidence from other malignancies remains useful as translational context rather than direct proof. In mixed solid tumors (non-GC data), whole-body PD-L1 imaging with [^89^Zr]Zr-DFO-atezolizumab correlated with treatment response and outperformed some tissue-based biomarkers (17). Similarly, in non-gastric tumor cohorts, [^89^Zr]Zr-DFO-pembrolizumab studies showed associations between tracer uptake, survival, and intra- and interlesional heterogeneity (18, 19).

### 4.2 Tumor microenvironment and stromal resistance phenotypes

Failure of immunotherapy in gastric cancer is often not explained by tumor-cell target loss alone. Instead, resistance commonly reflects an immunosuppressive microenvironment, stromal barriers, and immune exclusion. FAPI-based imaging is relevant because it visualizes fibroblast activation protein, a marker associated with activated cancer-associated fibroblasts and stromal remodeling.

Rong et al. reported that [^68^Ga]Ga-FAPI-04 PET/CT could noninvasively assess a cancer-associated fibroblast-rich immunosuppressive microenvironment in gastric cancer and was associated with outcome under PD-1 blockade (20). Higher FAPI uptake reflected a more immunosuppressive phenotype and was associated with reduced, not greater, benefit from PD-1 blockade. This makes FAPI imaging a resistance-oriented biomarker rather than a simple positive predictor of response. Xu et al. showed that [¹^8^F]FDG PET biomarkers obtained before and after neoadjuvant immunochemotherapy predicted postoperative pathological response in locally advanced gastric cancer (21). He et al. further found that, in recurrent gastric cancer treated with PD-1 inhibitors plus chemotherapy, pretreatment [¹^8^F]AlF-NOTA-FAPI-04 PET/CT parameters were significantly associated with progression-free and overall survival (22). Together, these findings suggest that FAPI-based imaging may support prognostic stratification and treatment decision-making.

Broader gastric-cancer imaging literature also supports noninvasive microenvironment assessment. Huang et al. showed that imaging-derived characterization of the tumor immune microenvironment correlated with response to immunotherapy in gastric cancer (23). Wang et al. likewise reported favorable diagnostic performance of FAPI-based imaging in a meta-analysis comparing FAPI and FDG imaging in gastric cancer, including improved lesion detection in several disease sites (24).

### 4.3 Effector-function imaging and early response assessment

If target imaging addresses whether treatment can “find” biologically relevant lesions, effector-function imaging addresses whether immune-mediated killing is actually occurring. Granzyme B PET is currently the most representative direction in this domain.

In gastric cancer, Liu et al. reported that [^68^Ga]Ga-NOTA-GSI PET/CT showed promising performance for early response prediction during combined immunotherapy, with an SUV_max-t threshold of 2.05 yielding sensitivity of 81.0% and specificity of 84.2% (25). However, these values were derived from *post hoc* ROC analysis and should therefore be regarded as exploratory rather than definitive. In a subsequent study, granzyme B PET also showed predictive value in microsatellite-stable gastric cancer receiving chemoimmunotherapy, suggesting that functional immune imaging may identify benefit even in less favorable subgroups (26).

The broader translational basis for granzyme B imaging is strengthened by two additional studies. Zhou et al. established [^68^Ga]Ga-grazytracer as a granzyme B-targeted PET probe capable of interrogating CD8^+^ T-cell effector function and predicting tumor response to immunotherapy in preclinical models and early clinical observations (27). Shen et al. then reported a phase 1/2 first-in-human trial of [^68^Ga]Ga-grazytracer PET in patients with solid tumors and lymphomas, demonstrating clinical feasibility for noninvasive assessment of immunotherapy response (28).

### 4.4 *In vivo* pharmacodynamic tracking

An important but underdeveloped application of immuno-PET in gastric cancer is *in vivo* pharmacodynamic tracking. Beyond baseline phenotyping, immuno-PET may reveal whether therapy is altering target biology *in vivo*.

In HER2-positive gastric cancer xenografts, Janjigian et al. showed that [^89^Zr]Zr-DFO-trastuzumab PET could monitor afatinib-induced biologic changes, illustrating how molecular imaging can capture treatment-related effects beyond conventional morphologic readouts (29). Although preclinical, this study remains relevant because it shows that immuno-PET may reflect therapy-induced modulation of target biology in real time.

More broadly, the expanding clinical literature on zirconium-89-based immuno-PET supports this concept. De Feo et al. reviewed the broader clinical role of [^89^Zr]Zr-DFO-immuno-PET in humans (non-GC data) and emphasized its value in assessing antibody biodistribution, pharmacokinetics, and biologic activity *in vivo* (30). He et al. also reported that [^89^Zr]Zr-DFO-KN035 immuno-PET could provide diagnosis and efficacy monitoring in patients with non-gastric PD-L1-positive solid malignancies, illustrating the potential for dynamic, lesion-level assessment of target expression and treatment effects (31).

### 4.5 Potential selection for targeted radionuclide therapy

PET imaging may eventually help identify candidates for targeted radionuclide therapy. At present, FAPI-based radioligand therapy is not established in gastric cancer, and this possibility should be framed cautiously. However, growing oncology evidence suggests that FAPI PET could evolve from a diagnostic and prognostic tool into a theranostic gateway for selected patients. In gastric cancer, this remains a forward-looking opportunity rather than current practice, but it is reasonable to acknowledge it as a relevant future direction (32) (**Table 1**).

**Table 1:** Representative studies of molecular PET and immuno-PET relevant to gastric cancer immunotherapy.

Study	Disease/setting	Study type	Sample size/level	Tracer/modality	Tracer format	Main finding	Relevance to GC immunotherapy
Chen et al., 2019	Gastric cancer	Clinical	64 patients	[¹^8^F]FDG PET/CT	Small molecule	FDG uptake correlated with PD-L1-related features.	Suggests conventional metabolic imaging may partly reflect immune biology.
Ibrahim et al.2024	Gastric cancer models	Preclinical	Cell lines/xenografts/PDX tissues	Anti-PD-L1 immuno-PET	Full-length antibody-based	PD-L1 expression was dynamic and heterogeneous; glycosylation affected imaging performance.	Supports biologic rationale, not clinical validation.
Hernandez et al.2023	Gastric adenocarcinoma	Clinical	8 patients	[^64^Cu]Cu-DOTA-trastuzumab PET/CT	Full-length antibody-based	HER2-targeted imaging was feasible but technically limited.	Proof of concept for lesion-level HER2 assessment.
Rong et al. 2022	GC receiving PD-1 blockade	Clinical	21 patients	[^68^Ga]Ga-FAPI-04 PET/CT	Small molecule	Higher FAPI uptake reflected an immunosuppressive CAF-rich phenotype and poorer PD-1 benefit.	Suggests stromal imaging may help resistance-oriented stratification.
Liu et al., 2024	GC receiving immunotherapy	Clinical	Exploratory cohort	[^68^Ga]Ga-NOTA-GSI PET/CT	Peptide-like tracer	Granzyme B PET showed early response-prediction potential.	Functional immune imaging may identify responders before RECIST change.
Liu et al., 2026	MSS gastric cancer	Clinical	Exploratory cohort	Granzyme B PET/CT	Peptide-like tracer	Predictive value extended to MSS disease.	May refine selection in less sensitive subgroups.
Xu et al. 2025	Locally advanced GC after neoadjuvant immunochemotherapy	Clinical	Exploratory cohort	[¹^8^F]FDG PET biomarkers	Small molecule	Baseline and post-treatment metrics predicted pathological response.	PET may help perioperative response stratification.
He et al. 2026	Recurrent GC receiving PD-1 inhibitor plus chemotherapy	Clinical	Exploratory cohort	[¹^8^F]A**l**F-NOTA-FAPI-04 PET/CT	Small molecule	Pretreatment FAPI-derived parameters correlated with survival.	FAPI imaging may contribute to prognostic stratification.

CAF, cancer-associated fibroblast; FDG, fluorodeoxyglucose; FAPI, fibroblast activation protein inhibitor; GC, gastric cancer; MSS, microsatellite stable; PDX, patient-derived xenograft; PET, positron emission tomography; RECIST, Response Evaluation Criteria in Solid Tumors.

## 5 PET in immune-related adverse events and perioperative risk management: current potential and gastric cancer-specific gaps

Direct gastric-cancer-specific evidence supporting PET for immune-related adverse event detection or perioperative risk management remains limited. Therefore, PET should currently be regarded as a hypothesis-generating adjunct rather than an established gastric-cancer-specific tool in these settings.

From a practical standpoint, FDG PET/CT may occasionally provide supportive information when unexpected inflammatory uptake complicates the distinction between residual disease, infection, and treatment-related inflammation (33). However, because FDG lacks specificity, abnormal uptake cannot automatically be equated with immune-related toxicity. Likewise, while perioperative immunotherapy is expanding in gastric cancer, evidence remains insufficient to conclude that PET can reliably determine surgical timing, predict postoperative complications, or guide perioperative risk stratification after neoadjuvant immunotherapy. This gap represents an important area for future gastric-cancer-focused investigation.

## 6 Discussion

The evidence base for molecular PET and immuno-PET in gastric cancer falls into three tiers: preclinical gastric cancer studies, early clinical gastric cancer studies, and translational evidence extrapolated from other tumor types (14–20, 25, 26, 29–31). This hierarchy matters because clinical maturity differs across tracers and applications (11, 12, 30). PD-L1-targeted imaging in gastric cancer remains supported mainly by preclinical work (14, 16), whereas HER2-targeted PET, FAPI PET, and granzyme B PET have begun to generate early clinical signals in gastric cancer cohorts (15, 20–26). None has yet achieved routine clinical implementation or prospective validation as a decision-changing biomarker in gastric cancer immunotherapy (11, 12, 30).

From a translational standpoint, the field is advancing in three main directions: effector-function imaging, particularly granzyme B PET, for early response assessment (25–28); stromal phenotyping, especially FAPI imaging, for identifying immunosuppressive or resistance-prone microenvironments (20, 22–24, 32); and whole-body target mapping, including HER2- and PD-L1-oriented approaches, for lesion-level phenotyping and potential pharmacodynamic monitoring (14–19, 29–31). Granzyme B and FAPI imaging may be closest to clinically useful stratification roles in gastric cancer, although both still require larger gastric-cancer-specific prospective validation (20–28).

Practical barriers remain substantial. Tracer format directly influences pharmacokinetics, imaging time, lesion contrast, radiation exposure, and workflow (11, 12, 16, 30). Intact antibodies offer high specificity but often require delayed imaging and may be less practical for perioperative decision-making or rapidly changing treatment settings (11, 16, 30). Smaller scaffold proteins or low-molecular-weight tracers allow faster imaging but may differ in biodistribution, background activity, and quantitative stability (11, 12, 16). In gastric cancer, where abdominal physiologic uptake, peritoneal disease, and lesion heterogeneity are common, these are direct barriers to broader clinical adoption (15, 20, 24).

Another challenge is evidence granularity. Some published findings in gastric cancer remain exploratory, including *post hoc* cutoffs for granzyme B PET and retrospective associations between FAPI-derived parameters and outcome (20–22, 25, 26). Several concepts discussed in this review, especially checkpoint imaging and pharmacodynamic monitoring, still rely partly on extrapolation from non-gastric malignancies (17–19, 30, 31). Such extrapolation is useful but should not be mistaken for direct gastric-cancer-specific validation (17–19, 31).

This review also has limitations. It is a narrative synthesis rather than a systematic review, and the included literature is heterogeneous in disease setting, tracer design, sample size, endpoints, and imaging timing (14, 15, 20–22, 25, 26, 29, 31). The analysis is primarily qualitative, and many cited studies are exploratory, single-center, or early phase (15, 20–22, 25, 26, 28, 31). Accordingly, this review should be viewed as a structured overview of an emerging field rather than a definitive evidence-based practice guide.

## 7 Conclusion

Molecular PET and immuno-PET are not yet standard tools in gastric cancer immunotherapy, but they offer a compelling framework for moving beyond static, single-site biomarkers. Their near-term value is most likely to lie in complementary roles, including whole-body target mapping, stromal phenotyping, early functional response assessment, and pharmacodynamic tracking within multimodal decision systems that also integrate pathology, endoscopy, and blood-based biomarkers. Future progress will depend on gastric-cancer-specific prospective validation, standardized quantification, and evidence that imaging-guided decisions can improve patient outcomes.
